# Assessment of Severe COVID-19 Outcomes Using Measures of Smoking Status and Smoking Intensity

**DOI:** 10.3390/ijerph18178939

**Published:** 2021-08-25

**Authors:** E. Melinda Mahabee-Gittens, Angelico Mendy, Ashley L. Merianos

**Affiliations:** 1 Division of Emergency Medicine, Cincinnati Children’s Hospital Medical Center, University of Cincinnati College of Medicine, Cincinnati, OH 45229, USA; 2 Division of Epidemiology, Department of Environmental and Public Health Sciences, University of Cincinnati College of Medicine, Cincinnati, OH 45267, USA; angelico.mendy@uc.edu; 3 School of Human Services, University of Cincinnati, Cincinnati, OH 45221, USA; ashley.merianos@uc.edu

**Keywords:** smoking, tobacco use, COVID-19, hospitalization, intensive care unit

## Abstract

Objective: Smoking status does not indicate the amount or length of tobacco use, and thus, it is an imperfect measure to assess the association between cigarette smoking and severe coronavirus disease 2019 (COVID-19) outcomes. This investigation assessed whether cigarette smoking status, intensity of smoking (i.e., average daily packs of cigarettes smoked), duration of smoking, and pack-years of smoking are associated with severe outcomes among adults diagnosed with COVID-19. Methods: We conducted a retrospective, cross-sectional study in which we identified consecutive patients diagnosed with COVID-19 at the University of Cincinnati healthcare system between 13 March 2020 and 30 September 2020 who had complete information on smoking status, severe COVID-19 outcomes, and covariates (i.e., demographics and comorbidities). We used logistic regression to evaluate the associations of smoking status and intensity of smoking with COVID-19 severity, defined as hospitalization, admission to intensive care unit (ICU), or death, adjusting for sociodemographics and comorbidities. Results: Among the 4611 COVID-19 patients included in the analysis, 18.2% were current smokers and 20.7% were former smokers. The prevalence of COVID-19 outcomes was 28.9% for hospitalization, 9.8% for ICU admission, and 1.4% for death. In the adjusted analysis, current smoking (AOR: 1.23, 95% CI: 1.02–1.49), former smoking (AOR: 1.28, 95% CI: 1.07–1.54), and pack-years of smoking (AOR: 1.09, 95% CI: 1.02–1.17) were associated with a higher prevalence of hospitalization. Average daily packs of cigarettes smoked was associated with a higher prevalence of hospitalization (AOR: 1.30, 95% CI: 1.10–1.53) and ICU admission (AOR: 1.23, 95% CI: 1.04–1.44). Conclusions: Smoking status, pack-years, and intensity of smoking were associated with hospitalizations in patients with COVID-19 and intensity of smoking was associated with ICU admission. The findings underscore the need for detailed information beyond smoking status when evaluating smokers with COVID-19 so that the potential for adverse sequelae may be optimally managed in at-risk patients.

## 1. Introduction

The toll of severe acute respiratory syndrome coronavirus 2 (SARS-CoV-2), the virus responsible for causing coronavirus disease 2019 (COVID-19), has resulted in staggering rates of hospitalizations and deaths across the U.S. [1]. Shortly after the onset of the COVID-19 pandemic, the World Health Organization determined that smokers were at risk for more severe COVID-19 outcomes compared to nonsmokers [2]. There are multiple reasons underlying smokers’ inherent risk to COVID-19-related adverse outcomes, including the effects of cigarette smoking on: the immune system [3], which renders smokers susceptible to viral and other infections [4,5]; airways and lungs, which results in increased airway secretions, impaired mucociliary clearance, increased permeability of airway epithelium, and airway inflammation [6]; and upregulation of the angiotensin-converting enzyme-2 (ACE2) receptor in both current and former smokers [7]. The COVID-19 spike protein specifically binds to the ACE-2 receptor and permits entry into cells; this association may facilitate an increased human-to-human spread of the virus and infection rates [8]. Moreover, many of the comorbidities that are associated with increased risk of severe COVID-19 outcomes are identical to those associated with tobacco use in smokers, including obesity, diabetes, asthma, chronic obstructive pulmonary disease (COPD), hypertension, chronic kidney disease (CKD), cardiovascular disease (CVD), and cancer [3,9]. 

Despite the strides made in COVID-19 vaccinations and research to combat the spread [10], it is believed that COVID-19 will eventually become an endemic infection [11]. Thus, more research is needed to identify specific risk factors of smokers so that clinicians can prioritize and aggressively treat those smokers more likely to experience severe COVID-19 outcomes. Prior research indicates that former and current smokers and smokers with higher cumulative smoking use are at risk for more severe COVID-19-related outcomes compared to never smokers [12,13,14]. However, the majority of these studies have been based on smoking status, which does not indicate the amount or length of tobacco use. Thus, smoking status is an imperfect measure to assess the associations between cigarette smoking and COVID-19 outcomes. Further, those studies have not examined detailed data on current and former smoking behavior, which may further elucidate severe outcomes. Thus, our objectives were to examine the associations of ever smoking status history, current and former cigarette smoking status, intensity of smoking (i.e., average daily packs of cigarettes smoked), duration of smoking, and pack-years of smoking with severe outcomes among adults diagnosed with COVID-19. Severe COVID-19-related outcomes were defined as hospitalization < 1 week following the diagnosis of a COVID-19 infection, intensive care unit (ICU) admission, and/or death during the hospital stay.

## 2. Methods

### Participants and Procedures

We conducted a retrospective, cross-sectional electronic medical record review of consecutive patients diagnosed with COVID-19 within the University of Cincinnati healthcare system (UC Health). UC Health includes clinics and hospitals in the metropolitan area of greater Cincinnati, which has a population of approximately two million people [15]. Patients were eligible if they were diagnosed with COVID-19 at UC Health between 13 March 2020 and 30 September 2020 and had complete information on smoking status, severe COVID-19 outcomes, and covariates (i.e., demographics and comorbidities, see Section 3.3). Patients were ineligible if they had missing data on smoking status. We identified 15,995 patients with COVID-19 diagnoses. After the exclusion of 11,384 patients with missing smoking status data, 4611 participants were included in our study. Compared to patients who were included, patients who were excluded due to missing data on smoking status were younger, female, had a lower prevalence of comorbidities, and had a lower prevalence of severe COVID-19 outcomes (see Supplementary Table S1). The UC institutional review board (IRB) exempted this study from review since we used de-identified data which did not include Health Insurance Portability and Accountability Act (HIPAA) identifiers.

## 3. Measures

### 3.1. Tobacco Use

Electronic medical records were abstracted to obtain self-reported tobacco use measures: ever smoker status history (never smoker, ever smoker), current and former smoking status history (never smoker, current smoker, former smoker), intensity of smoking (average daily packs of cigarettes smoked), duration of smoking (number of years of smoking), and pack-years of smoking. Pack-years of smoking was calculated as number of packs of cigarettes smoked per day multiplied by number of years. It was analyzed as a continuous variable and as a categorical variable with the following groups (1–10, 11–20, 21–30, >30 cigarette packs).

### 3.2. Severe COVID-19 Outcomes

The following were classified as severe COVID-19 outcomes: (a) hospitalization for ≥24 h, defined as admission to a hospital within the UC healthcare system < 1 week following the diagnosis of a COVID-19 infection; COVID-19 was again confirmed upon admission; (b) intensive care unit (ICU) admission as opposed to admission to a regular floor; and (c) death during the hospital stay.

### 3.3. Covariates

We included the following self-reported sociodemographics as covariates: sex, race/ethnicity, and smoking status. Age at COVID-19 diagnosis was calculated using patients’ date of birth. The following concurrent comorbidities, defined using the International Statistical Classification of Diseases and Related Health Problems [16] (ICD-10) codes, were considered covariates: obesity (E66), diabetes (E11), asthma (J45), COPD (J44), hypertension (I10), CKD (N18), CVD (I00–I99), and neoplasm or history of neoplasm (C00–D49).

### 3.4. Statistical Analysis

Descriptive analyses were conducted to summarize the characteristics of the study participants overall and by demographics, smoking status, comorbidities, and severe COVID-19 outcomes. *p*-values for differences across these characteristics were estimated using chi-square tests. The associations of cigarette smoking status (i.e., ever smoker history, former and current smoking status), packs smoked per day (i.e., intensity of smoking), years of smoking (i.e., duration of smoking), and pack-years of smoking with severe COVID-19 outcomes were assessed using logistic regression models to estimate the adjusted odds ratios (AORs) and corresponding 95% confidence intervals (95% CIs). The models were adjusted for sociodemographic characteristics (i.e., age, sex, race/ethnicity) and comorbidities (e.g., obesity). The dose–response relationships between average daily packs of cigarettes smoked and severe COVID-19 outcomes were assessed using restricted cubic splines of the adjusted prevalence of COVID-19 outcomes as a function of intensity of smoking, with three knots using the command *adjustrcspline* in STATA 17.0 [17]. The analyses were performed in SAS Version 9.4 [18], and two-sided *p*-values < 0.05 were considered statistically significant. This paper is reported following the STROBE statement [19].

## 4. Results

### 4.1. Description of Study Population

The 4611 patients with COVID-19 diagnoses included in our study had a median age of 48.1 years (IQR: 29.7, 63.6), and 63.1% were female (Table 1). In total, 61% were never smokers, 18.2% were current smokers, and 20.7% were former smokers. The most common comorbidities were hypertension (41.5%), CVD (37.4%), and cancer history (25.7%). The prevalence of COVID-19 outcomes was 28.9% for hospitalization, 9.8% for ICU admission, and 1.4% for death during the hospital stay. See Table 1 for other patient characteristics.

### 4.2. Characteristics of Study Participants by Smoking Status

Compared to never smokers, ever smokers and former smokers tended to: be older, male, non-Hispanic Black, have a higher prevalence of all comorbidities, and have a higher prevalence of severe COVID-19 outcomes (Table 2). Compared to never smokers, COVID-19 patients who were current smokers were more likely to: be younger, male, non-Hispanic Black, and have a higher prevalence of all comorbidities, with the exception of obesity and CKD. Current smokers also had a higher prevalence of hospitalization and ICU admission.

Given the high prevalence of comorbidities in the study sample, we examined the association of comorbidities and severe COVID-19 outcomes. In adjusted logistic regression analyses, the odds of hospitalization were higher for all comorbidities except for obesity and asthma, and the odds for ICU admission were higher for all comorbidities except for obesity, asthma, hypertension, and cancer. The odds of death were higher in those with a history of CKD or CVD (see Table 3). 

### 4.3. Smoking Status and Severe COVID-19 Outcomes

In adjusted logistic regression analyses, the odds of hospitalization were higher in ever smokers (AOR: 1.26, 95% CI: 1.08, 1.46), current smokers (AOR: 1.23, 95% CI: 1.02, 1.49), and former smokers (AOR: 1.28, 95% CI: 1.07, 1.54) compared to never smokers (Table 4). The increase in the odds of hospitalization among former smokers remained significant after accounting for multiple comparison (i.e., *p*-value < 0.025, which corresponded to the ratio of the 0.05 significance level over the number of comparisons for the former and current smoking status variables). The odds of hospitalization were also increased with number of packs smoked per day (AOR: 1.30, 95% CI: 1.10, 1.53) and pack-years of smoking (AOR: 1.09, 95% CI: 1.02, 1.17), but not with years of smoking (AOR: 1.00, 95% CI: 0.99, 1.01). In addition to being associated with hospitalization, number of packs smoked per day was associated with ICU admission (AOR: 1.23, 95% CI: 1.04, 1.44). None of the smoking variables were associated with death (see Table 4). 

**Figure 1:**
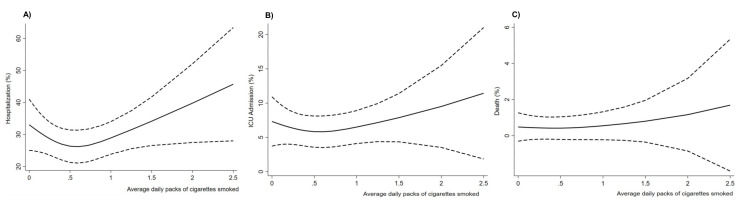
Restricted cubic splines for the associations of average daily packs of cigarettes smoked with hospitalization (**A**), ICU admission (**B**), and death (**C**). Solid lines show the smoothed adjusted prevalence, and the dashed lines indicate 95% confidence intervals. The graphs suggest a positive exposure–response relationship for hospitalization and ICU admission at daily packs of cigarettes smoked ≥ 0.5 but no association with death.

We characterized the dose–response for the associations between average daily packs of cigarettes smoked and severe COVID-19 outcomes using restricted cubic splines. As illustrated in Figure 1, the splines demonstrated a positive exposure–response relationship of average daily packs of cigarettes smoked with hospitalization (Figure 1A) and ICU admission (Figure 1B) but not with death (Figure 1C). Increases in the prevalence of hospitalization and ICU admission were apparent at average daily packs of cigarettes smoked ≥ 0.5. 

## 5. Discussion

The results of this study provide further evidence that current and former smoking status, as well as ever smoking status, intensity of smoking, and pack-years, are risk factors for severe COVID-19 outcomes. Current smokers were up at 1.2 increased risk and former smokers were at 1.3 increased risk of being hospitalized compared to never smokers; pack-years of smoking was also associated with a higher prevalence of hospitalization. The intensity of smoking, or average number of packs of cigarettes smoked daily, was associated with higher hospitalization and ICU admission. These findings are congruent to those of recent systematic reviews and meta-analyses that have reported similar associations with smoking status and increased risk of COVID-19-related hospitalizations, ICU admissions, and deaths [7,13,20]. Similar to prior work, we also observed a high prevalence of severe outcomes in both former smokers and current smokers. It is possible that former smokers remain at risk for continued COVID-19-related sequelae due to the potential modulatory effects of smoking on ACE2 protein expression in former and current smokers since ACE2 serves as a possible receptor by which COVID-19 may enter into epithelial cells [21,22]. Further, it is well-established that former smokers remain at risk for many illnesses, including lung cancer, CVD, and metabolic syndrome [23,24,25]. While quitting smoking greatly decreases this risk, it does not abolish the risk. Additionally, former smokers may have had longer exposure to tobacco smoke pollutants, which places them at increased risk of associated comorbidities [26] and, potentially, COVID-19. Finally, current smokers may underreport their smoking status, which may result in apparent higher rates of COVID-19-related morbidity compared to those who report that they are never smokers [7]. 

Our results also indicated that patients in this sample were at increased risk of hospitalization per every 10 pack-years increase. These results suggest that there are dose–response effects associated with smoking history and lifetime exposure to cigarette smoking. Similar results were also observed in a previous study that reported that patients who smoked more than 30 pack-years had 2.25 and 1.89 higher odds of hospitalization and death following a COVID-19 diagnosis, respectively, compared to never smokers [14]. These findings underscore the need for more detailed information beyond smoking status when evaluating smokers with COVID-19 so that the potential for adverse sequelae may be anticipated and optimally managed in at-risk patients. 

The limitations of this study should be noted. First, patients were from a single healthcare system in the Midwestern U.S. state of Ohio, and thus, results may not be generalizable to patients in the U.S. as a whole, by U.S. States, or within Ohio. Second, since this study was cross-sectional, causality or timing between smoking and COVID-19 severity cannot be established. Third, since smoking status and smoking behavior were self-reported, the prevalence of current smokers and the number of pack-years of smoking may have been higher than reported. However, these underestimates would likely only strengthen our observed associations. Fourth, we excluded patients who did not have information on their smoking status in their electronic medical records. Those excluded may not have wanted to disclose this information, which may indicate that they were current or former smokers, or they may not have been asked due to their clinical presentation, which required immediate treatment. Therefore, the sample of participants included in our study may not be representative of all COVID-19 patients diagnosed at UC Health. Participants with data on smoking had a higher prevalence of comorbidities and more severe COVID-19 than those without information on smoking. Lastly, in order to be included in this study sample, patients had to have a COVID-19 positive test, and those who had a positive test may have been more ill than those who did not or those who were not tested. Nevertheless, this study provides important information about COVID-19 outcomes among a large, racially/ethnically diverse sample of COVID-19-positive adult patients who had a high prevalence of current and former smoking. 

## 6. Conclusions

The results of this study suggest that current and former smoking status alone is an imperfect measure by which to assess a patient’s risk of severe outcomes from COVID-19. Therefore, we recommend that smoking status, intensity of smoking, and pack-years should be assessed when examining patients who smoke who also have a COVID-19 diagnosis. Future studies should ask these questions in real-time upon COVID-19 diagnosis to supplement the smoking history in electronic medical records that may be missing, outdated, not detailed, or inaccurate [27,28]. By including detailed, real-time tobacco use assessments, smokers with COVID-19 diagnoses can be given proactive counseling about their tobacco use and optimally treated for potentially severe outcomes. 

## Figures and Tables

**Table 1:** Overall characteristics of study participants (*N* = 4611).

Characteristics	*N* (%)
Older adults (65 + years old)	1119 (24.7)
Female sex	2911 (63.1)
Race/ethnicity	
Non-Hispanic White	2557 (55.5)
Non-Hispanic Black	1526 (33.1)
Hispanic	269 (5.8)
Other	259 (5.6)
Smoking	
Never	2815 (61.0)
Current	841 (18.2)
Former	955 (20.7)
Comorbidities	
Obesity	1010 (21.9)
Type 2 diabetes	942 (20.4)
Asthma	666 (14.4)
COPD	437 (9.5)
Hypertension	1913 (41.5)
CKD	492 (10.7)
CVD	1724 (37.4)
Cancer history	1187 (25.7)
COVID-19 outcomes	
Hospitalization	1333 (28.9)
ICU admission	450 (9.8)
Death	66 (1.4)

Abbreviations: COPD: chronic obstructive pulmonary disease; CKD: chronic kidney disease; CVD: cardiovascular disease; ICU: intensive care unit.

**Table 2:** Characteristics of study participants by smoking status (*N* = 4611).

	Never	Ever Smokers	*p*-Value ^†^	Current Smokers	*p*-Value ^‡^	Former Smokers	*p*-Value ^⸸^
Older adults (65 or older)	582 (20.7)	537 (29.9)	**0.001**	142 (16.9)	**0.02**	395 (41.4)	**<0.001**
Female sex	1871 (66.5)	1040 (57.9)	**<0.001**	480 (57.1)	**<0.001**	560 (58.6)	**<0.001**
Race/ethnicity			**<0.001**		**<0.001**		**<0.001**
Non-Hispanic White	1558 (55.3)	999 (55.6)		442 (52.6)		557 (58.3)	
Non-Hispanic Black	830 (29.5)	696 (38.8)		349 (41.5)		347 (36.3)	
Hispanic	228 (8.1)	41 (2.3)		20 (2.4)		21 (2.2)	
Other	199 (7.1)	60 (3.3)		30 (3.6)		30 (3.1)	
Comorbidities							
Obesity	552 (19.6)	458 (25.5)	**<0.001**	156 (18.5)	0.49	302 (31.6)	**<0.001**
Type 2 diabetes	452 (16.1)	490 (27.7)	**<0.001**	174 (20.7)	**0.002**	316 (33.1)	**<0.001**
Asthma	346 (12.3)	320 (17.8)	**<0.001**	137 (16.3)	**0.003**	183 (19.2)	**<0.001**
COPD	81 (2.9)	356 (19.8)	**<0.001**	155(18.4)	**<0.001**	201 (21.0)	**<0.001**
Hypertension	910 (32.3)	1003 (55.8)	**<0.001**	388 (46.1)	**<0.001**	615 (64.4)	**<0.001**
CKD	213 (7.6)	279 (15.5)	**<0.001**	81 (9.6)	0.05	198 (20.7)	**<0.001**
CVD	836 (29.7)	888 (49.4)	**<0.001**	332 (39.5)	**<0.001**	556 (58.2)	**<0.001**
Cancer history	606 (21.5)	581 (32.3)	**<0.001**	202 (24.0)	0.13	379 (39.7)	**<0.001**
Severe COVID-19 outcomes							
Hospitalization	677 (24.0)	656 (36.5)	**<0.001**	268 (31.9)	**<0.001**	388 (40.6)	**<0.001**
ICU admission	234 (8.3)	216 (12.0)	**<0.001**	95 (11.3)	**0.008**	121 (12.7)	**<0.001**
Death	26 (0.9)	40 (2.2)	**<0.001**	10 (1.2)	0.49	30 (3.1)	**<0.001**

Abbreviations: COPD: chronic obstructive pulmonary disease; CKD: chronic kidney disease; CVD: cardiovascular disease; ICU: intensive care unit. ^†^ *p*-values comparing characteristics between current or former smokers and never smokers. ^‡^ *p*-values comparing characteristics between current smokers and never smokers. ^⸸^ *p*-values comparing characteristics between former smokers and never smokers. *p*-values calculated using chi-square tests. Bold indicates significant associations, *p* < 0.05.

**Table 3:** Association of comorbidities with severe COVID-19 outcomes.

	Hospitalization AOR (95% CI)	ICU Admission AOR (95% CI)	Death AOR (95% CI)
Obesity	0.99 (0.83, 1.19)	0.85 (0.66, 1.11)	0.56 (0.31, 1.05)
Diabetes	**1.57 (1.30, 1.89)**	**1.51 (1.16, 1.97)**	1.39 (0.80, 2.42)
Asthma	0.94 (0.76, 1.16)	0.82 (0.61, 1.12)	1.25 (0.65, 2.39)
COPD	**1.76 (1.38, 2.4)**	**1.59 (1.17, 2.17)**	1.57 (0.86, 2.89)
Hypertension	**1.32 (1.08, 1.60)**	1.02 (0.75, 1.38)	1.72 (0.73, 4.07)
CKD	**2.05 (1.63, 2.58)**	**1.74 (1.30, 2.33)**	**1.87 (1.07, 3.27)**
CVD	**2.78 (2.33, 3.31)**	**3.52 (2.68, 4.63)**	**8.35 (2.99, 23.34)**
Cancer	**0.80 (0.68, 0.95)**	0.79 (0.62, 1.01)	0.92 (0.55, 1.56)

Abbreviations: COPD, chronic obstructive pulmonary disease; CKD, chronic kidney disease. Models mutually adjusted for age, sex, race/ethnicity, and comorbidities: obesity, type 2 diabetes, asthma, COPD, hypertension, CKD, CVD, and cancer or history of cancer. Bold indicates significant associations, *p* < 0.05.

**Table 4:** Adjusted odds ratios for the associations of smoking with severe COVID-19 outcomes.

	Hospitalization AOR (95% CI)	ICU Admission AOR (95% CI)	Death AOR (95% CI)
Ever smoker status			
Never	Reference	Reference	Reference
Ever	**1.26 (1.08, 1.46)**	1.20 (0.96, 1.49)	1.15 (0.65, 2.00)
Former and current smoking status			
Never	Reference	Reference	Reference
Current	**1.23 (1.02, 1.49)**	1.24 (0.94, 1.64)	0.77 (0.34, 1.71)
Former	**1.28 (1.07, 1.54) ^¶^**	1.16 (0.89, 1.51)	1.34 (0.75, 2.40)
Packs smoked per day	**1.30 (1.10, 1.53)**	**1.23 (1.04, 1.44)**	0.99 (0.56, 1.73)
Years of smoking	1.00 (0.99, 1.01)	1.00 (0.99, 1.01)	1.01 (0.99, 1.03)
Pack-years of smoking			
Per 10 pack-years	**1.09 (1.02, 1.17)**	1.07 (0.99, 1.15)	1.00 (0.86, 1.16)
Never smokers	Reference	Reference	Reference
1 to 10 pack-years	0.87 (0.64, 1.18)	0.87 (0.56, 1.34)	N/A
11 to 20 pack-years	1.07 (0.73, 1.56)	0.92 (0.53, 1.58)	N/A
21 to 30 pack-years	1.21 (0.80, 1.85)	0.81 (0.42, 1.53)	N/A
>31 pack-years	1.44 (0.97, 2.15)	1.22 (0.71, 2.09)	N/A

Abbreviations: AOR, adjusted odds ratio; CI, confidence interval; N/A, not applicable, odds not computed due to the small number of deaths. Models adjusted for age, sex, race/ethnicity, and comorbidities (obesity, type 2 diabetes, asthma, COPD, hypertension, CKD, CVD, and cancer or history of cancer). Bold indicates significant associations, *p* < 0.05. **^¶^** Indicates significant association for former and current smoking status after adjustment for multiple comparison.

## Data Availability

The dataset will be made available to other researchers upon request for the purpose of reproducing the findings.

## Supplementary Materials

The following are available online at https://www.mdpi.com/article/10.3390/ijerph18178939/s1, Table S1: Characteristics of participants with and without missing data on smoking status.
