# Supplementary material for: Assessment of Severe COVID-19 Outcomes Using Measures of Smoking Status and Smoking Intensity
Source: Int J Environ Res Public Health. 2021 Aug 25;18(17):8939. doi: 10.3390/ijerph18178939 (PMC8431679; doi:10.3390/ijerph18178939)
Supplement: Supplementary file 1 [file ijerph-18-08939-s001.zip › ijerph-1272815-supplementary.pdf]

<u>Supplementary Table S1</u>: Characteristics of participants with and without missing data on smoking status

Characteristics	Participants without missing data N (%)	Participants with missing data N (%)	P-value
**Older adults (65+ years old)**	1,119 (24.7)	2,277 (5.2)	**< 0.001**
**Female sex**	2,911 (63.1)	8,278 (72.6)	**< 0.001**
**Race/ethnicity**			**< 0.001**
Non-Hispanic White	2,557 (55.5)	7,386 (64.9)	
Non-Hispanic Black	1,526 (33.1)	2,542 (22.3)	
Hispanic	269 (5.8)	2.71 (2.3)	
Other	259 (5.6)	1,184 (10.4)	
**Smoking**			
Never	2,815 (61.0)	N/A	
Current	841 (18.2)	N/A	
Former	955 (20.7)	N/A	
**Comorbidities**			
Obesity	1,010 (21.9)	1,013 (8.8)	**< 0.001**
Type 2 diabetes	942 (20.4)	834 (7.3)	**< 0.001**
Asthma	666 (14.4)	651 (5.7)	**< 0.001**
COPD	437 (9.5)	340 (3.0)	**< 0.001**
Hypertension	1,913 (41.5)	1,768 (15.5)	**< 0.001**
CKD	492 (10.7)	428 (3.8)	**< 0.001**
CVD	1,724 (37.4)	1,605 (14.1)	**< 0.001**
Cancer history	1,187 (25.7)	1,196 (10.5)	**< 0.001**
**Severe COVID-19 outcomes**			
Hospitalization	1,333 (28.9)	966 (8.5)	**< 0.001**
ICU admission	450 (9.8)	271 (2.4)	**< 0.001**
Death	66 (1.4)	67 (0.6)	**< 0.001**

Abbreviations: COPD: chronic obstructive pulmonary disease; CKD: chronic kidney disease; CVD: cardiovascular disease; ICU: intensive care unit. Bold indicates significant associations, $p < 0.05$.
